# Synthesis and structure of a binuclear calcium nitrate coordination complex with bridging zwitterionic nicotinic acid

**DOI:** 10.1107/S2056989026001271

**Published:** 2026-02-13

**Authors:** Gulmira Burkitbaeva, Zulfiya Djumanazarova, Aysanem Bektursinova, Jamshid Ashurov, Shakhnoza Kadirova, Odil Choriev, Saule Meldebekova, Batirbay Torambetov

**Affiliations:** a Karakalpak State University, 1 Ch.Abdirov St. Nukus, 230112, Uzbekistan; b Institute of Bioorganic Chemistry, Academy of Sciences of Uzbekistan, M., Ulugbek, St, 83, Tashkent, 100125, Uzbekistan; c National University of Uzbekistan named after Mirzo Ulugbek 4 University St Tashkent 100174 Uzbekistan; University of Aberdeen, United Kingdom

**Keywords:** crystal structure, calcium, nicotinic acid, Hirshfeld surface analysis

## Abstract

The title coordination complex, bis­(μ-pyridin-1-ium-3-carboxyl­ato-κ^2^*O*:*O*′)bis­[di­aqua­bis­(nitrato-κ^2^*O*,*O*′)calcium(II)], [Ca_2_(C_6_H_5_NO_2_)_2_(NO_3_)_4_(H_2_O)_4_], was prepared from calcium nitrate and nicotinic acid in a water–ethanol solvent mixture. The asymmetric unit contains a half mol­ecule of the complex, with the calcium atom exhibiting a coordination number of eight, forming a distorted dodeca­hedral geometry with a mixed-ligand environment. The μ_2_-O,*O*′ bridging zwitterionic nicotinic acid mol­ecules generate a centrosymmetric dinuclear complex. The extended structure features N—H⋯O, O—H⋯O and C—H⋯O hydrogen bonds, which generate a three-dimensional network. Hirshfeld surface and two-dimensional fingerprint plot analyses were performed to qu­antify and visualize the inter­molecular inter­actions contributing to the overall cohesion of the structure.

## Abstract 2

The crystal structure of [Ca_2_(C_6_H_5_NO_2_)_2_(H_2_O)_2_(NO_3_)_2_] reveals an eight-coordinate distorted dodeca­hedral Ca^II^ center with mixed ligands. A Hirshfeld surface analysis elucidated the inter­molecular inter­actions consolidating the crystal.

## 1. Chemical context

Nicotinic acid (C_6_H_5_NO_2_; niacin or vitamin B_3_) is an essential nutrient that plays a crucial role in human metabolism. It is used as a dietary supplement and is also utilized therapeutically for the management of coronary heart diseases (Malik & Kashyap, 2003 ▸). Clinically, nicotinic acid is primarily prescribed to regulate elevated cholesterol levels and provides several additional pharmacological benefits (Carlson, 2005 ▸). Furthermore, it has been demonstrated to decrease the incidence and severity of cardiovascular events as well as overall mortality (Canner *et al.*, 1986 ▸). Structurally, nicotinic acid contains a nitro­gen atom within the pyridine ring and a carb­oxy­lic acid (–COOH) functional group, allowing it to coordinate with metal ions through multiple donor sites (Zhou & Wang, 2015 ▸; Cherkasova *et al.*, 2018 ▸). Under certain conditions, the pyridine nitro­gen atom can become protonated, while the carb­oxy­lic acid group loses a proton, resulting in the formation of the zwitterionic form of the ligand (Iqbal *et al.*, 2021 ▸). In the present study, we describe the utilization of nicotinic acid in the synthesis of a coordination complex of calcium, [Ca_2_(C_6_H_5_NO_2_)_2_(H_2_O)_4_(NO_3_)_4_], (**I**).
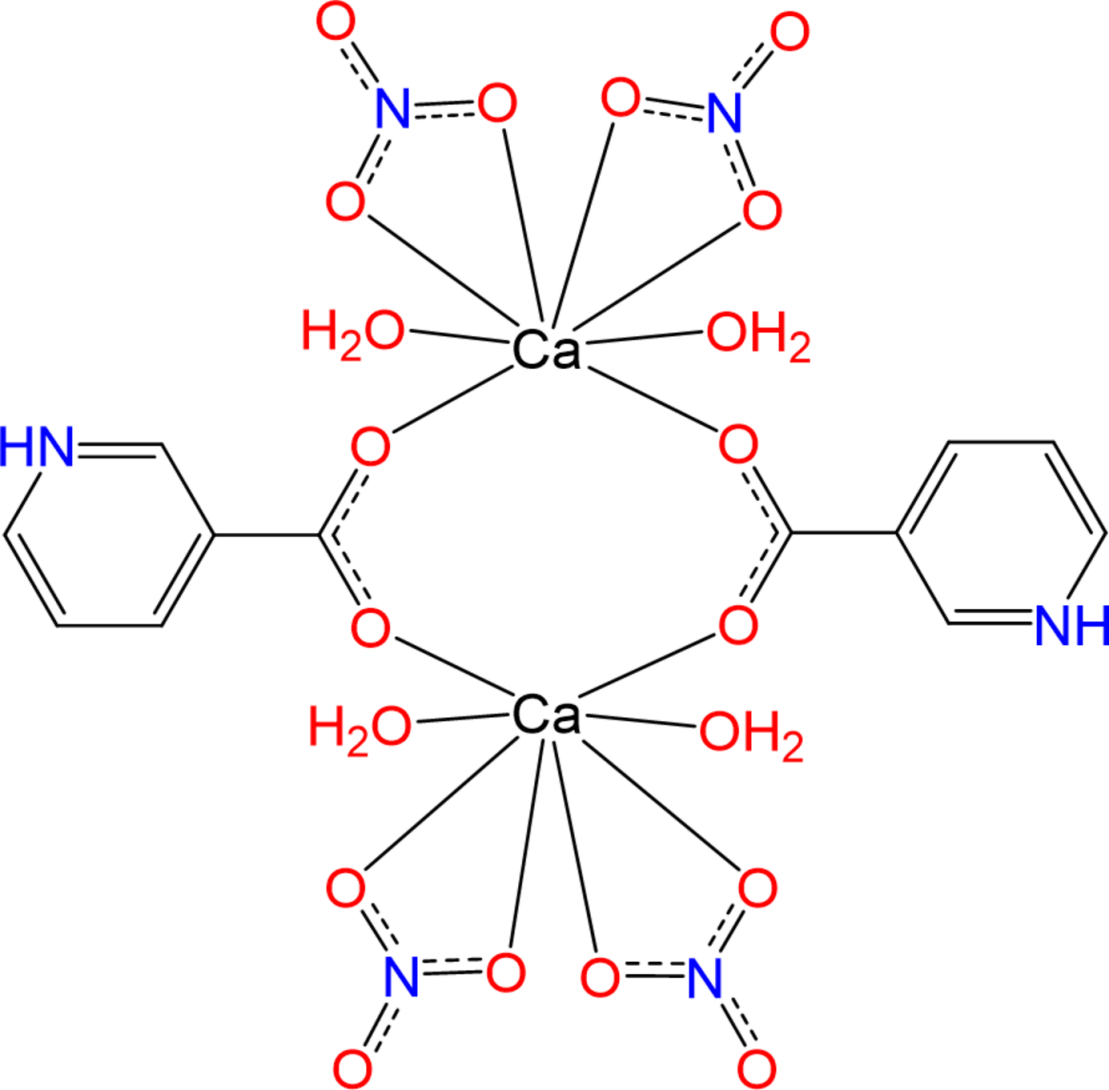


## 2. Structural commentary

**Figure 1:**
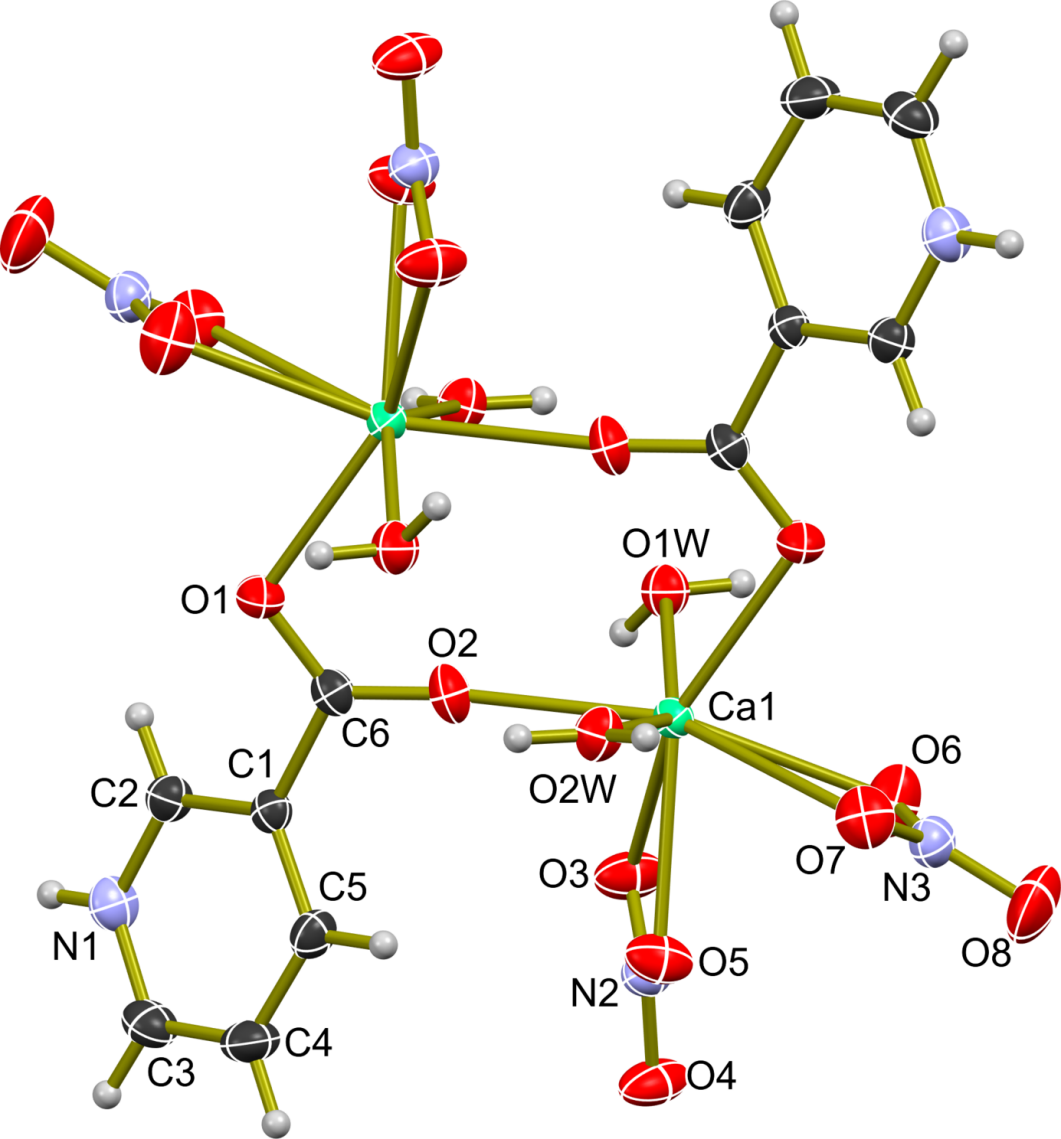
The mol­ecular structure of (**I**) with displacement ellipsoids shown at the 50% probability level. Symmetry code (i) 1 − *x*, 1 − *y*, −*z*.

Single-crystal X-ray diffraction analysis revealed that (**I**) crystallizes in the triclinic system in space group *P*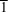. The asymmetric unit contains half of the complex, in which the central calcium atom is coordinated by two nitrate anions in a bidentate fashion, two aqua ligands, and two unidentate carboxyl­ate O atoms, resulting in a coordination number of eight and forming a distorted dodeca­hedral geometry with a mixed-ligand structure. The nicotinate ligand exists in its zwitterionic form, where the pyridine nitro­gen atom N1 is protonated and the deprotonated carboxyl­ate group [C6—O1 = 1.259 (3) Å. C6—O2 = 1.241 (3) Å, O1—C6—O2 = 123.94 (19)°] coordinates through both oxygen atoms to two calcium centers [Ca1⋯Ca1^i^ = 4.3188 (5) Å; symmetry code: (i) 1 − *x*, 1 − *y*, −*z*] thereby acting as a μ_2_-*O*,*O′* bridging ligand (Fig. 1 ▸). The Ca—O bond lengths (Table 1 ▸) for the carboxyl­ate ligands (O1 and O2) are the shortest, indicating the strongest coordination to the metal centre. The Ca—O bonds for the aqua ligands (O1*W* and O2*W*) are slightly longer, while the longest Ca—O bonds (O3, O5, O6, O7) are observed in the bidentate nitrate ligands, where electron delocalization in the N—O bonds of the nitrate ions reduces the donor ability of oxygen atoms and weakens the Ca—O inter­actions.

## 3. Supra­molecular features

**Figure 2:**
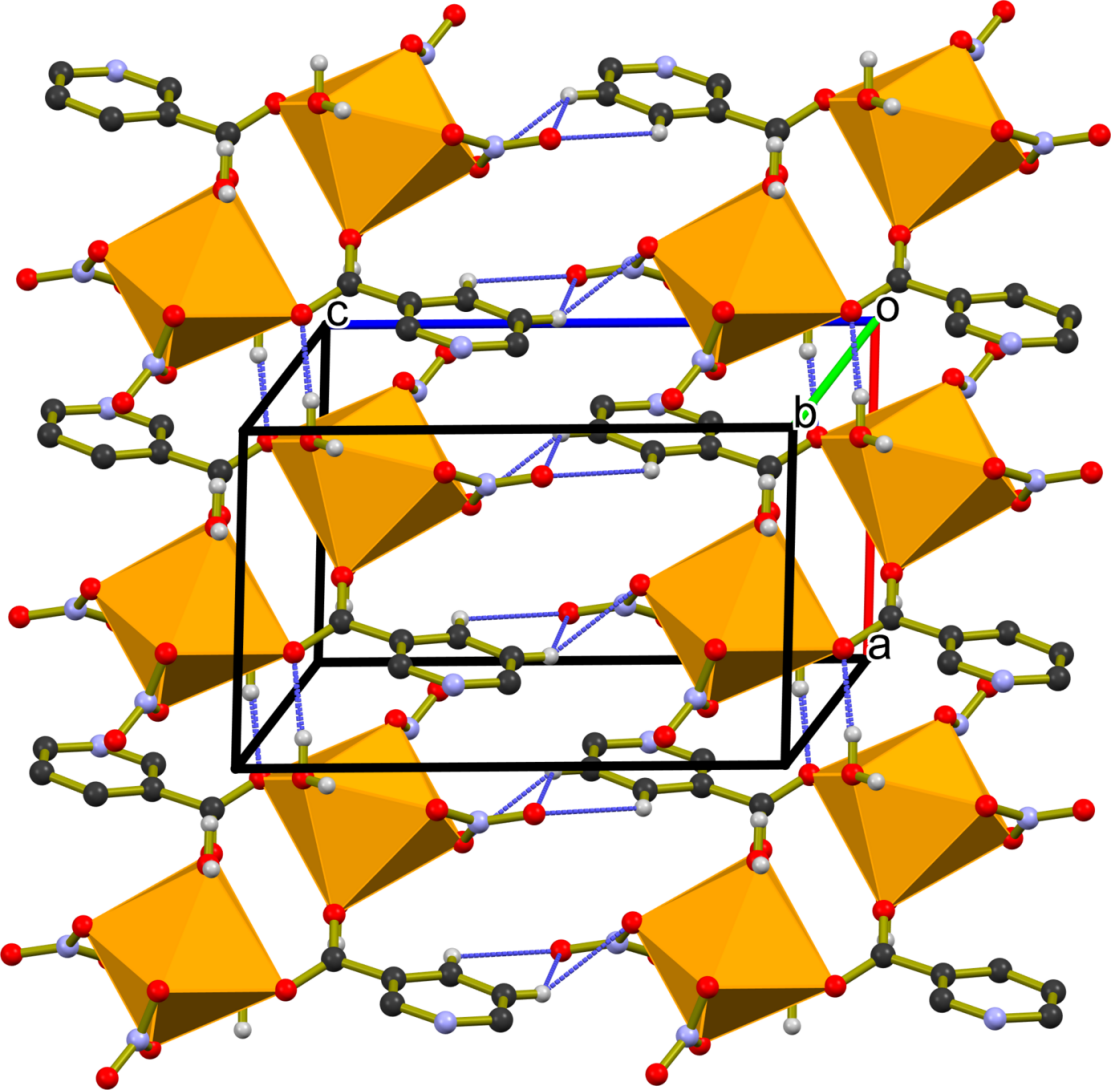
Visualization of the packing in (**I**) viewed along the *b-*axis direction, showing the CaO_8_ moieties and polyhedra and C—H⋯O, O—H⋯O and N–H⋯O inter­actions as blue dashed lines.

As a result of the presence of numerous acceptor oxygen atoms, the title complex exhibits various hydrogen-bonding inter­actions, including N—H⋯O, O—H⋯O and C—H⋯O types (Table 2 ▸). The mol­ecular packing viewed along the *b*-axis direction (Fig. 2 ▸) reveals that two mol­ecular units of the complex are connected through O—H⋯O inter­actions extending along the *a*-axis direction. Along the *b*-axis, the mol­ecules are further linked through C—H⋯O and O—H⋯O inter­actions. The coordinated water mol­ecules also participate in hydrogen bonding with oxygen atoms from the nitrate anions and the carboxyl­ate groups, as well as with phenyl C—H groups, forming O—H⋯O and C—H⋯O linkages (Table 2 ▸). Along the *c*-axis direction, the crystal packing features N—H⋯O and several C—H⋯O inter­actions, involving the nitrate oxygen atoms as acceptors and phenyl or amine hydrogen atoms as donors. The C—H⋯O inter­actions involving the nitrate oxygens and aromatic C—H groups form a one-dimensional assembly along the *c*-axis direction. Collectively, these hydrogen-bonding inter­actions generate a three-dimensional supra­molecular network.

## 4. Hirshfeld Surface Analysis

**Figure 3:**
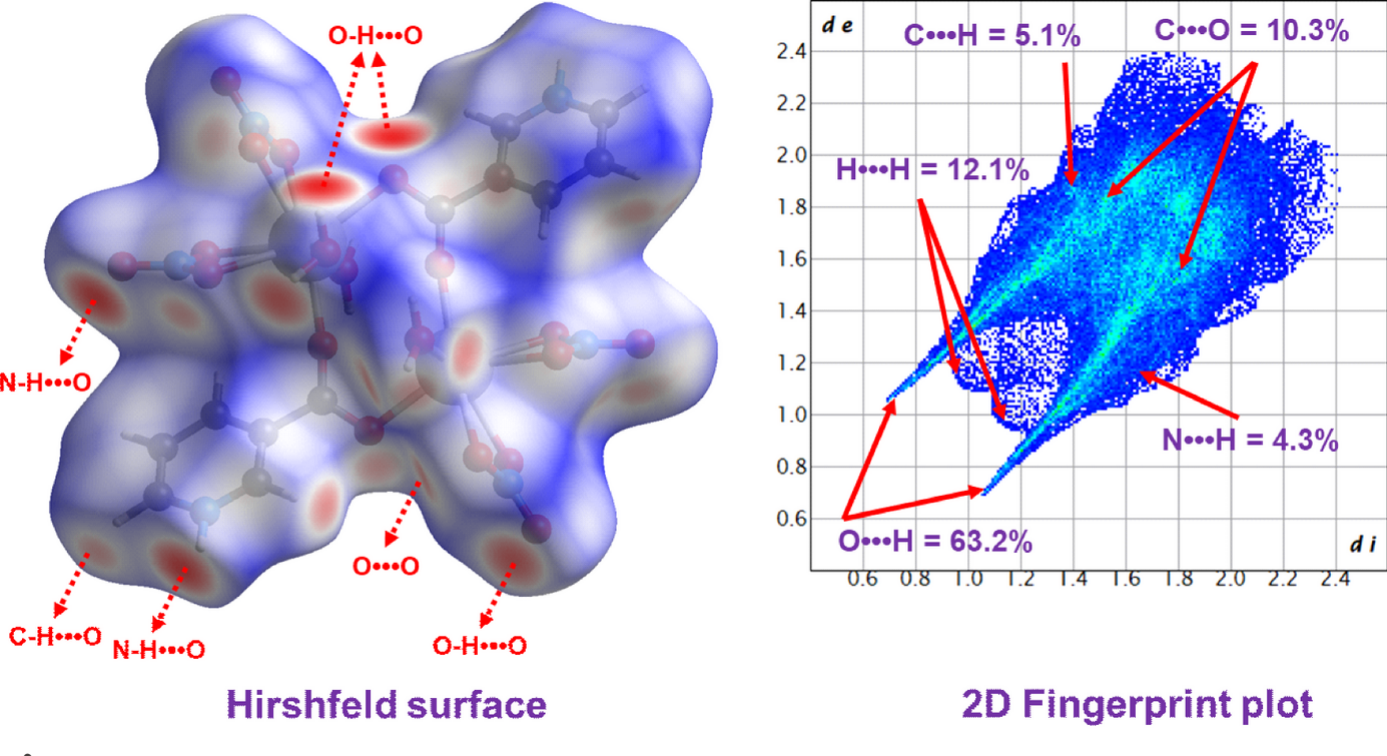
The Hirshfeld surface and corresponding two-dimensional fingerprint plots for (**I**).

The inter­molecular inter­actions contributing to the stability of the complex were analyzed using Hirshfeld surface and fingerprint plot analysis implemented in *CrystalExplorer* (Spackman *et al.*, 2021 ▸). The analysis revealed that O⋯H/H⋯O inter­actions are predominant, constituting 63.2% of the total inter­molecular contacts. This predominance arises from the abundance of oxygen atoms derived from nitrate anions, coordinated water mol­ecules, and carboxyl­ate groups. These inter­actions are visualized as intense red spots on the Hirshfeld surface. Other notable inter­actions include N⋯H/H⋯N (4.3%), C⋯H/H⋯C (5.1%), H⋯H (12.1%), and C⋯O/O⋯C (10.3%) contacts, together accounting for approximately 95% of the total surface inter­actions. Additionally, the O⋯H/H⋯O inter­actions appear as two sharp spikes at *d*_e_ + *d*_i_ = 1.8 Å in the corresponding fingerprint plot (Fig. 3 ▸).

## 5. Database survey

A survey of the Cambridge Structural Database (CSD, Version 6.01, November 2025; Groom *et al.*, 2016 ▸) revealed 209 crystal structures containing nicotinic acid ligands exhibiting an O_2_ coordination mode. Among these, thirteen structures feature the ligand in a zwitterionic form, wherein the pyridine nitro­gen atom is protonated while coordination to the metal occurs through deprotonated carboxyl­ate oxygen atoms (O_2_ coordination set). The reported metal atoms in such complexes include Sc^+3^ (refcode AFIMIO, Cherkasova *et al.*, 2018 ▸), Cr^+3^ (BONTED, Gonzalez-Vergara *et al.*, 1982 ▸), Eu^+3^ (DEYLOJ, Lu *et al.*, 2007 ▸; XOYQIM, Kong *et al.*, 2009 ▸), Ce^+3^ (DEYLUP, Lu *et al.*, 2007 ▸), Fe^+3^ (INOBET, Chen, 2010 ▸; SINZAS, Chen *et al.*, 2013 ▸), Mo^+6^ (IZASUX, Chen *et al.*, 2004 ▸; JEPNEX, Cotton *et al.*, 1990 ▸), Al^+3^ (RIWCUZ, RIWCUZ01, Zhao *et al.*, 2024 ▸), U^+6^ (SUZREN, Andreev *et al.*, 2020 ▸), and Gd^+3^ (XOYQOS, Kong *et al.*, 2009 ▸). Thus it may be seen that no crystal structure containing calcium ions coordinated by a zwitterionic nicotinic acid ligand has been reported to date.

## 6. Synthesis and crystallization

Ca(NO_3_)_2_·4H_2_O (0.236 g, 1.00 mmol) was added under continuous stirring to a solution of nicotinic acid (0.123 g, 1.00 mmol) dissolved in 10 ml of ethanol–water (50:50). The resulting colorless solution was stirred for 3 h and was then left to stand at room temperature. After two weeks, colorless blocks suitable for X-ray diffraction were obtained (yield 70%) by the slow evaporation of the solvent.

## 7. Refinement details

Crystal data, data collection and structure refinement details are summarized in Table 3 ▸. H atoms were positioned geometrically (O—H = 0.85–0.88, N—H = 0.96, C—H = 0.93 Å) and refined as riding with *U*_iso_(H) = 1.2*U*_eq_(N, C) or 1.5*U*_eq_(O).

## Supplementary Material

Crystal structure: contains datablock(s) I. DOI: 10.1107/S2056989026001271/hb8192sup1.cif

Structure factors: contains datablock(s) I. DOI: 10.1107/S2056989026001271/hb8192Isup2.hkl

CCDC reference: 2529092

Additional supporting information:  crystallographic information; 3D view; checkCIF report

## Figures and Tables

**Table 1:** Selected bond lengths (Å)

Ca1—O1^i^	2.4020 (15)	Ca1—O3	2.5810 (16)
Ca1—O1*W*	2.4426 (15)	Ca1—O5	2.4990 (16)
Ca1—O2	2.3314 (15)	Ca1—O6	2.5116 (17)
Ca1—O2*W*	2.3927 (15)	Ca1—O7	2.6680 (17)

Symmetry code: (i) 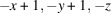.

**Table 2:** Hydrogen-bond geometry (Å, °)

*D*—H⋯*A*	*D*—H	H⋯*A*	*D*⋯*A*	*D*—H⋯*A*
O1*W*—H1*WA*⋯O8^ii^	0.85	2.14	2.955 (3)	160
O1*W*—H1*WB*⋯O1^iii^	0.85	1.88	2.723 (2)	173
O2*W*—H2*WA*⋯O1*W*^i^	0.88	2.35	2.887 (2)	119
O2*W*—H2*WB*⋯O1^iv^	0.88	2.43	3.268 (2)	158
O2*W*—H2*WB*⋯O7^v^	0.88	2.32	2.897 (2)	123
N1—H1⋯O4^vi^	0.86	1.98	2.782 (2)	155
C2—H2⋯O2*W*^ii^	0.93	2.40	3.327 (2)	175
C3—H3⋯O3^vi^	0.93	2.45	3.228 (3)	141
C4—H4⋯O4^vii^	0.93	2.46	3.102 (3)	127

Symmetry codes: (i) 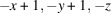; (ii) 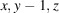; (iii) 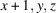; (iv) 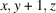; (v) 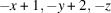; (vi) 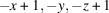; (vii) 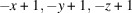.

**Table 3:** Experimental details

Crystal data	
Chemical formula	[Ca_2_(C_6_H_5_NO_2_)_2_(NO_3_)_4_(H_2_O)_4_]
*M*_r_	646.48
Crystal system, space group	Triclinic, *P* 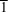
Temperature (K)	293
*a*, *b*, *c* (Å)	7.2785 (2), 7.7034 (2), 11.8102 (2)
α, β, γ (°)	76.204 (2), 88.236 (2), 67.828 (2)
*V* (Å^3^)	594.24 (3)
*Z*	1
Radiation type	Cu *K*α
μ (mm^−1^)	5.19
Crystal size (mm)	0.3 × 0.2 × 0.12

Data collection	
Diffractometer	XtaLAB Synergy, Single source at home/near, HyPix3000
Absorption correction	Multi-scan (*CrysAlis PRO*; Rigaku OD, 2023 ▸)
*T*_min_, *T*_max_	0.522, 1.000
No. of measured, independent and observed [*I* > 2σ(*I*)] reflections	5079, 2278, 2204
*R*_int_	0.025
(sin θ/λ)_max_ (Å^−1^)	0.615

Refinement	
*R*[*F*^2^ > 2σ(*F*^2^)], *wR*(*F*^2^), *S*	0.036, 0.096, 1.09
No. of reflections	2278
No. of parameters	183
H-atom treatment	H-atom parameters constrained
Δρ_max_, Δρ_min_ (e Å^−3^)	0.38, −0.50

Computer programs: *CrysAlis PRO* (Rigaku OD, 2023 ▸), *SHELXT2018/2* (Sheldrick, 2015*a* ▸), *SHELXL2016/6* (Sheldrick, 2015*b* ▸) and *OLEX2* (Dolomanov *et al.*, 2009 ▸).

## supplementary crystallographic information

### Bis(µ-pyridin-1-ium-3-carboxylato-κ2O:O')bis[diaqua\ bis(nitrato-κ2O,O')calcium(II)] Crystal data

**Table:**

[Ca_2_(C_6_H_5_NO_2_)_2_(NO_3_)_4_(H_2_O)_4_]	*Z* = 1
*M**_r_* = 646.48	*F*(000) = 332
Triclinic, *P*1	*D*_x_ = 1.807 Mg m^−^^3^
*a* = 7.2785 (2) Å	Cu *K*α radiation, λ = 1.54184 Å
*b* = 7.7034 (2) Å	Cell parameters from 4225 reflections
*c* = 11.8102 (2) Å	θ = 3.9–71.3°
α = 76.204 (2)°	µ = 5.19 mm^−^^1^
β = 88.236 (2)°	*T* = 293 K
γ = 67.828 (2)°	Block, colourless
*V* = 594.24 (3) Å^3^	0.3 × 0.2 × 0.12 mm

### Bis(µ-pyridin-1-ium-3-carboxylato-κ2O:O')bis[diaqua\ bis(nitrato-κ2O,O')calcium(II)] Data collection

**Table:**

XtaLAB Synergy, Single source at home/near, HyPix3000 diffractometer	2278 independent reflections
Radiation source: micro-focus sealed X-ray tube, PhotonJet (Cu) X-ray Source	2204 reflections with *I* > 2σ(*I*)
Mirror monochromator	*R*_int_ = 0.025
Detector resolution: 10.0000 pixels mm^-1^	θ_max_ = 71.5°, θ_min_ = 3.9°
ω scans	*h* = −8→8
Absorption correction: multi-scan (CrysAlisPro; Rigaku OD, 2023)	*k* = −9→9
*T*_min_ = 0.522, *T*_max_ = 1.000	*l* = −14→11
5079 measured reflections	

### Bis(µ-pyridin-1-ium-3-carboxylato-κ2O:O')bis[diaqua\ bis(nitrato-κ2O,O')calcium(II)] Refinement

**Table:**

Refinement on *F*^2^	Primary atom site location: dual
Least-squares matrix: full	Hydrogen site location: mixed
*R*[*F*^2^ > 2σ(*F*^2^)] = 0.036	H-atom parameters constrained
*wR*(*F*^2^) = 0.096	*w* = 1/[σ^2^(*F*_o_^2^) + (0.0521*P*)^2^ + 0.3004*P*] where *P* = (*F*_o_^2^ + 2*F*_c_^2^)/3
*S* = 1.09	(Δ/σ)_max_ < 0.001
2278 reflections	Δρ_max_ = 0.38 e Å^−^^3^
183 parameters	Δρ_min_ = −0.50 e Å^−^^3^
0 restraints	

### Bis(µ-pyridin-1-ium-3-carboxylato-κ2O:O')bis[diaqua\ bis(nitrato-κ2O,O')calcium(II)] Special details

**Table:**

Geometry. All esds (except the esd in the dihedral angle between two l.s. planes) are estimated using the full covariance matrix. The cell esds are taken into account individually in the estimation of esds in distances, angles and torsion angles; correlations between esds in cell parameters are only used when they are defined by crystal symmetry. An approximate (isotropic) treatment of cell esds is used for estimating esds involving l.s. planes.

### Bis(µ-pyridin-1-ium-3-carboxylato-κ2O:O')bis[diaqua\ bis(nitrato-κ2O,O')calcium(II)] Fractional atomic coordinates and isotropic or equivalent isotropic displacement parameters (Å^2^)

**Table:**

	*x*	*y*	*z*	*U*_iso_*/*U*_eq_	
Ca1	0.65567 (5)	0.56954 (5)	0.12434 (3)	0.02000 (14)	
O1	0.2555 (2)	0.2795 (2)	0.05968 (12)	0.0302 (3)	
O1W	0.8866 (2)	0.2683 (2)	0.08421 (13)	0.0281 (3)	
H1WA	0.893487	0.174012	0.141145	0.042*	
H1WB	1.003606	0.268073	0.082744	0.042*	
O2	0.4355 (2)	0.4144 (2)	0.12536 (14)	0.0324 (4)	
O2W	0.3380 (2)	0.8281 (2)	0.07100 (13)	0.0291 (3)	
H2WA	0.220459	0.819656	0.071424	0.044*	
H2WB	0.312208	0.952976	0.046846	0.044*	
O3	0.7743 (3)	0.3173 (2)	0.32194 (14)	0.0393 (4)	
O4	0.7352 (3)	0.3969 (2)	0.48750 (13)	0.0438 (5)	
O5	0.5874 (3)	0.6076 (2)	0.32756 (13)	0.0364 (4)	
O6	0.9434 (3)	0.6251 (2)	0.19867 (17)	0.0413 (4)	
O7	0.6916 (2)	0.8917 (2)	0.14747 (15)	0.0370 (4)	
O8	0.9511 (3)	0.8834 (3)	0.2375 (2)	0.0575 (6)	
N1	0.2536 (3)	−0.0327 (3)	0.39757 (16)	0.0298 (4)	
H1	0.245919	−0.144565	0.412136	0.036*	
N2	0.6989 (3)	0.4398 (3)	0.38014 (15)	0.0287 (4)	
N3	0.8640 (3)	0.8012 (3)	0.19526 (16)	0.0290 (4)	
C1	0.3034 (3)	0.2220 (3)	0.26437 (17)	0.0204 (4)	
C2	0.2854 (3)	0.0452 (3)	0.28886 (18)	0.0250 (4)	
H2	0.295230	−0.019107	0.230181	0.030*	
C3	0.2331 (4)	0.0548 (3)	0.48484 (19)	0.0326 (5)	
H3	0.207827	−0.003366	0.558899	0.039*	
C4	0.2493 (4)	0.2298 (4)	0.46462 (19)	0.0343 (5)	
H4	0.234927	0.292264	0.524625	0.041*	
C5	0.2876 (3)	0.3141 (3)	0.35361 (18)	0.0267 (4)	
H5	0.302616	0.431917	0.339274	0.032*	
C6	0.3352 (3)	0.3123 (3)	0.14110 (17)	0.0223 (4)	

### Bis(µ-pyridin-1-ium-3-carboxylato-κ2O:O')bis[diaqua\ bis(nitrato-κ2O,O')calcium(II)] Atomic displacement parameters (Å^2^)

**Table:**

	*U*^11^	*U*^22^	*U*^33^	*U*^12^	*U*^13^	*U*^23^
Ca1	0.0215 (2)	0.0201 (2)	0.0190 (2)	−0.00918 (16)	0.00170 (15)	−0.00375 (15)
O1	0.0331 (8)	0.0423 (9)	0.0198 (7)	−0.0199 (7)	0.0031 (6)	−0.0070 (6)
O1W	0.0244 (7)	0.0268 (8)	0.0319 (8)	−0.0090 (6)	0.0007 (6)	−0.0060 (6)
O2	0.0316 (8)	0.0311 (8)	0.0384 (9)	−0.0195 (7)	0.0072 (7)	−0.0036 (7)
O2W	0.0283 (8)	0.0235 (7)	0.0343 (8)	−0.0089 (6)	−0.0003 (6)	−0.0065 (6)
O3	0.0587 (11)	0.0265 (8)	0.0288 (8)	−0.0102 (8)	0.0019 (7)	−0.0094 (6)
O4	0.0831 (14)	0.0328 (9)	0.0200 (8)	−0.0297 (9)	−0.0021 (8)	−0.0014 (6)
O5	0.0499 (10)	0.0243 (8)	0.0262 (8)	−0.0077 (7)	0.0056 (7)	−0.0015 (6)
O6	0.0329 (9)	0.0264 (8)	0.0605 (11)	−0.0073 (7)	−0.0070 (8)	−0.0084 (8)
O7	0.0322 (9)	0.0299 (8)	0.0406 (9)	−0.0067 (7)	−0.0033 (7)	−0.0013 (7)
O8	0.0685 (14)	0.0548 (12)	0.0676 (13)	−0.0425 (11)	−0.0096 (11)	−0.0156 (10)
N1	0.0372 (10)	0.0196 (8)	0.0311 (9)	−0.0131 (8)	0.0017 (8)	0.0002 (7)
N2	0.0429 (11)	0.0241 (9)	0.0218 (8)	−0.0177 (8)	0.0019 (8)	−0.0022 (7)
N3	0.0337 (10)	0.0277 (9)	0.0287 (9)	−0.0166 (8)	0.0015 (8)	−0.0041 (7)
C1	0.0177 (9)	0.0211 (9)	0.0215 (9)	−0.0067 (7)	0.0001 (7)	−0.0043 (7)
C2	0.0273 (10)	0.0221 (10)	0.0257 (10)	−0.0095 (8)	0.0024 (8)	−0.0062 (8)
C3	0.0379 (12)	0.0354 (12)	0.0220 (10)	−0.0149 (10)	0.0036 (9)	−0.0012 (9)
C4	0.0470 (14)	0.0368 (12)	0.0228 (10)	−0.0180 (11)	0.0031 (9)	−0.0107 (9)
C5	0.0326 (11)	0.0227 (10)	0.0279 (10)	−0.0134 (9)	−0.0003 (8)	−0.0071 (8)
C6	0.0191 (9)	0.0203 (9)	0.0248 (10)	−0.0060 (7)	0.0026 (7)	−0.0032 (7)

### Bis(µ-pyridin-1-ium-3-carboxylato-κ2O:O')bis[diaqua\ bis(nitrato-κ2O,O')calcium(II)] Geometric parameters (Å, º)

**Table:**

Ca1—O1^i^	2.4020 (15)	O3—N2	1.248 (2)
Ca1—O1W	2.4426 (15)	O4—N2	1.242 (2)
Ca1—O2^i^	3.0060 (17)	O5—N2	1.254 (2)
Ca1—O2	2.3314 (15)	O6—N3	1.249 (2)
Ca1—O2W	2.3927 (15)	O7—N3	1.255 (3)
Ca1—O3	2.5810 (16)	O8—N3	1.234 (3)
Ca1—O5	2.4990 (16)	N1—H1	0.8600
Ca1—O6	2.5116 (17)	N1—C2	1.335 (3)
Ca1—O7	2.6680 (17)	N1—C3	1.336 (3)
Ca1—N2	2.9335 (17)	C1—C2	1.379 (3)
Ca1—N3	3.0034 (18)	C1—C5	1.384 (3)
Ca1—C6^i^	3.055 (2)	C1—C6	1.508 (3)
O1—C6	1.259 (3)	C2—H2	0.9300
O1W—H1WA	0.8508	C3—H3	0.9300
O1W—H1WB	0.8506	C3—C4	1.361 (3)
O2—C6	1.241 (3)	C4—H4	0.9300
O2W—H2WA	0.8821	C4—C5	1.388 (3)
O2W—H2WB	0.8820	C5—H5	0.9300
			
O1^i^—Ca1—O1W	84.73 (5)	Ca1—O2W—H2WA	127.6
O1^i^—Ca1—O2^i^	46.53 (4)	Ca1—O2W—H2WB	127.8
O1^i^—Ca1—O3	147.44 (6)	H2WA—O2W—H2WB	104.6
O1^i^—Ca1—O5	142.29 (6)	N2—O3—Ca1	93.43 (12)
O1^i^—Ca1—O6	81.75 (6)	N2—O5—Ca1	97.23 (12)
O1^i^—Ca1—O7	72.20 (5)	N3—O6—Ca1	100.57 (13)
O1W—Ca1—O2^i^	73.14 (5)	N3—O7—Ca1	92.80 (12)
O1W—Ca1—O3	72.44 (5)	C2—N1—H1	118.5
O1W—Ca1—O5	122.30 (5)	C2—N1—C3	123.07 (19)
O1W—Ca1—O6	89.72 (5)	C3—N1—H1	118.5
O1W—Ca1—O7	133.97 (5)	O3—N2—Ca1	61.43 (10)
O2—Ca1—O1^i^	119.02 (5)	O3—N2—O5	118.50 (17)
O2—Ca1—O1W	81.19 (5)	O4—N2—Ca1	171.39 (15)
O2—Ca1—O2^i^	72.65 (5)	O4—N2—O3	120.76 (19)
O2—Ca1—O2W	75.77 (5)	O4—N2—O5	120.74 (19)
O2—Ca1—O3	80.76 (6)	O5—N2—Ca1	57.68 (10)
O2—Ca1—O5	92.75 (6)	O6—N3—Ca1	55.29 (10)
O2—Ca1—O6	156.07 (6)	O6—N3—O7	117.38 (18)
O2—Ca1—O7	144.83 (6)	O7—N3—Ca1	62.53 (11)
O2W—Ca1—O1^i^	84.04 (5)	O8—N3—Ca1	172.48 (16)
O2W—Ca1—O1W	145.16 (5)	O8—N3—O6	121.4 (2)
O2W—Ca1—O2^i^	75.22 (5)	O8—N3—O7	121.2 (2)
O2W—Ca1—O3	127.68 (6)	C2—C1—C5	118.86 (18)
O2W—Ca1—O5	84.95 (5)	C2—C1—C6	119.50 (17)
O2W—Ca1—O6	120.97 (5)	C5—C1—C6	121.63 (18)
O2W—Ca1—O7	72.40 (5)	N1—C2—C1	119.38 (19)
O3—Ca1—O2^i^	139.04 (5)	N1—C2—H2	120.3
O3—Ca1—O7	107.41 (5)	C1—C2—H2	120.3
O5—Ca1—O2^i^	157.64 (5)	N1—C3—H3	120.2
O5—Ca1—O3	50.05 (5)	N1—C3—C4	119.6 (2)
O5—Ca1—O6	73.43 (6)	C4—C3—H3	120.2
O5—Ca1—O7	70.09 (5)	C3—C4—H4	120.3
O6—Ca1—O2^i^	125.81 (5)	C3—C4—C5	119.4 (2)
O6—Ca1—O3	75.39 (6)	C5—C4—H4	120.3
O6—Ca1—O7	48.68 (5)	C1—C5—C4	119.71 (19)
O7—Ca1—O2^i^	112.33 (5)	C1—C5—H5	120.1
C6—O1—Ca1^i^	109.07 (12)	C4—C5—H5	120.1
Ca1—O1W—H1WA	109.4	O1—C6—Ca1^i^	48.00 (10)
Ca1—O1W—H1WB	109.5	O1—C6—C1	117.01 (17)
H1WA—O1W—H1WB	104.5	O2—C6—Ca1^i^	75.99 (12)
Ca1—O2—Ca1^i^	107.35 (5)	O2—C6—O1	123.94 (19)
C6—O2—Ca1^i^	80.40 (12)	O2—C6—C1	119.05 (18)
C6—O2—Ca1	170.25 (14)	C1—C6—Ca1^i^	164.88 (13)
			
Ca1^i^—O1—C6—O2	2.9 (2)	C2—N1—C3—C4	−1.6 (4)
Ca1^i^—O1—C6—C1	−177.59 (13)	C2—C1—C5—C4	−1.6 (3)
Ca1^i^—O2—C6—O1	−2.24 (19)	C2—C1—C6—Ca1^i^	25.7 (6)
Ca1^i^—O2—C6—C1	178.29 (17)	C2—C1—C6—O1	32.5 (3)
Ca1—O3—N2—O4	−170.42 (18)	C2—C1—C6—O2	−148.0 (2)
Ca1—O3—N2—O5	8.8 (2)	C3—N1—C2—C1	1.7 (3)
Ca1—O5—N2—O3	−9.1 (2)	C3—C4—C5—C1	1.7 (4)
Ca1—O5—N2—O4	170.07 (18)	C5—C1—C2—N1	−0.1 (3)
Ca1—O6—N3—O7	−7.8 (2)	C5—C1—C6—Ca1^i^	−153.0 (4)
Ca1—O6—N3—O8	171.91 (19)	C5—C1—C6—O1	−146.1 (2)
Ca1—O7—N3—O6	7.25 (19)	C5—C1—C6—O2	33.4 (3)
Ca1—O7—N3—O8	−172.5 (2)	C6—C1—C2—N1	−178.77 (18)
N1—C3—C4—C5	−0.2 (4)	C6—C1—C5—C4	177.1 (2)

Symmetry code: (i) −*x*+1, −*y*+1, −*z*.

### Bis(µ-pyridin-1-ium-3-carboxylato-κ2O:O')bis[diaqua\ bis(nitrato-κ2O,O')calcium(II)] Hydrogen-bond geometry (Å, º)

**Table:**

*D*—H···*A*	*D*—H	H···*A*	*D*···*A*	*D*—H···*A*
O1*W*—H1*WA*···O8^ii^	0.85	2.14	2.955 (3)	160
O1*W*—H1*WB*···O1^iii^	0.85	1.88	2.723 (2)	173
O2*W*—H2*WA*···O1*W*^i^	0.88	2.35	2.887 (2)	119
O2*W*—H2*WB*···O1^iv^	0.88	2.43	3.268 (2)	158
O2*W*—H2*WB*···O7^v^	0.88	2.32	2.897 (2)	123
N1—H1···O4^vi^	0.86	1.98	2.782 (2)	155
C2—H2···O2*W*^ii^	0.93	2.40	3.327 (2)	175
C3—H3···O3^vi^	0.93	2.45	3.228 (3)	141
C4—H4···O4^vii^	0.93	2.46	3.102 (3)	127

Symmetry codes: (i) −*x*+1, −*y*+1, −*z*; (ii) *x*, *y*−1, *z*; (iii) *x*+1, *y*, *z*; (iv) *x*, *y*+1, *z*; (v) −*x*+1, −*y*+2, −*z*; (vi) −*x*+1, −*y*, −*z*+1; (vii) −*x*+1, −*y*+1, −*z*+1.
